# Sensitivity and negative predictive value of sentinel lymph node biopsy for cutaneous melanoma for diagnosing nodal metastasis: meta-analysis of diagnostic test accuracy

**DOI:** 10.1093/bjsopen/zraf089

**Published:** 2025-08-14

**Authors:** Conrad Harrison, Samuel Willis, Mary Rose Harvey, Rakhshan Kamran, Ryckie G Wade, Thomas D Dobbs, Oliver Cassell

**Affiliations:** Nuffield Department of Orthopaedics, Rheumatology and Musculoskeletal Sciences, University of Oxford, Oxford, UK; Department of Plastic and Reconstructive Surgery, Oxford University Hospitals, Oxford, UK; Department of Medicine, Oxford University Clinical Academic Graduate School, Oxford, UK; Swansea University Medical School, Swansea University, Swansea, UK; Department of Medicine, Oxford University Clinical Academic Graduate School, Oxford, UK; Nuffield Department of Orthopaedics, Rheumatology and Musculoskeletal Sciences, University of Oxford, Oxford, UK; Leeds Institute for Medical Research, University of Leeds, Leeds, UK; Swansea University Medical School, Swansea University, Swansea, UK; Welsh Centre for Burns and Plastic Surgery, Morriston Hospital, Swansea, UK; Department of Plastic and Reconstructive Surgery, Oxford University Hospitals, Oxford, UK

## Abstract

**Background:**

Sentinel lymph node biopsy provides information about disease staging and the need for adjuvant therapy. The consequences of a false-negative result are potentially severe. The risk of a false-negative result should be quantified. The aims of this study were to estimate the sensitivity of sentinel lymph node biopsy based on studies following up patients for at least a mean or median of 5 years, appraise the risk of bias, and provide negative predictive value estimates across a range of pretest probabilities.

**Methods:**

Ovid MEDLINE and Embase databases were searched from inception to 28 May 2025. Studies were screened independently and in duplicate, with a third author resolving conflicts. All original comparative and non-comparative English language research studies were included if the sensitivity of sentinel lymph node biopsy was calculable and participants had been followed up for a mean or median of 5 years. Risk of bias was assessed using the Quality Assessment of Diagnostic Accuracy Studies 2 tool. Sensitivity estimates were calculated and pooled by random-effects meta-analysis. A negative predictive value curve was plotted using the pooled sensitivity estimate and a range of plausible pretest probabilities.

**Results:**

Fourteen studies with 8447 patients were included. The pooled sensitivity estimate was 0.85 (95% confidence interval 0.80 to 0.88). The negative predictive value estimates fell between 0.93 and 0.97, depending on pretest probability. Existing negative predictive value estimates are at risk of positive bias.

**Conclusion:**

Sentinel lymph node biopsy is a sensitive test used to rule out lymph node metastasis in cutaneous melanoma. Clinicians can use negative predictive value estimates to counsel patients about the probability of false-negative results, for example, by offering reassurance to patients with thin melanomas and negative sentinel lymph node biopsy.

## Teaser

Sentinel lymph node biopsy (SLNB) results provide important information about the need for adjuvant therapy, and therefore the risk of a false-negative result should be quantified. A negative predictive value (NPV) curve was plotted using the pooled sensitivity estimate and a range of plausible pretest probabilities. Clinicians can use these NPV estimates to counsel patients about the probability of false-negative results, for example, by offering reassurance to patients with thin melanomas and a negative SLNB.

## Introduction

Sentinel lymph node biopsy (SLNB) is commonly performed to diagnose lymph node metastases in cutaneous melanoma^1^. The results of SLNB contribute to disease staging, assisting clinical teams in planning adjuvant therapy and follow-up^2^. They also provide important prognostic information for the patient and their family in terms of disease recurrence and overall survival^3^. In some instances, SLNB can produce false-negative results, defined as a nodal recurrence in a lymph node basin that has previously been biopsied with a negative result^4^. Because SLNB positivity often determines the need for adjuvant therapy, a false-negative result can result in undiagnosed metastatic disease that is potentially left to progress. Clinicians and policymakers should take the accuracy of SLNB into account when interpreting its results, and patients must be counselled about the risks of a false-negative biopsy. Ideally, this risk would be quantified. It may be reasonable to intensify follow-up in patients with higher risk of false-negative results, and it may be helpful to tell patients the probability that their test has provided a true result.

Previous studies have attempted to quantify the risk of a false-negative SLNB, but there are important limitations in the literature to date. First, there is inconsistency in the terminology and statistics used to describe the accuracy of SLNB for cutaneous melanoma. Many studies report ‘false-negative rates’, although the definition of this statistic varies between authors, with some choosing to divide the number of false-negatives by the sum of false-negatives and true-positives, and others dividing the number of false-negatives by the total sample size^4^. Terms such as sensitivity (which has a near universally accepted definition as the number of true-positives divided by the sum of true-positives and false-negatives) would avoid confusion here^5^.

Arguably, a more clinically relevant statistic is the negative predictive value (NPV): the probability that a patient's negative result is true. In keeping with the principles of Bayesian statistics, the NPV of a test varies with the pretest probability of any given result^6–8^. According to this principle, a negative SLNB result may be more likely to be true for a low-risk melanoma than a high-risk one. This is consistent with observational studies^9–11^ finding that tumours leading to false-negative SLNB results are thicker, on average, than those leading to true-negative results. It is also consistent with the results of the MSLT-I trial^12^, which showed a higher proportion of false-negative results in patients with melanomas of thickness > 3.5 mm than those with tumours of intermediate thickness (1.2–3.5 mm). Although this nuance has generally not been applied in previous NPV calculations for SLNB^4^, it is favoured in assessments of diagnostic test accuracy across other fields^13^.

In 2011, Valsecchi *et al*.^14^ pooled false-negative rates (false-negatives divided by the sum of true-positives and false-negatives) across 71 studies, resulting in an estimate of 13%. However, at the time, the risk of bias in these pooled estimates had not been evaluated with purpose-built tools. The Quality Assessment of Diagnostic Accuracy Studies (QUADAS) 2 framework^15^ is now widely used to evaluate the risk and direction of bias in studies of diagnostic test accuracy, although QUADAS-2 domains are seldom considered in the evaluation of SLNB for cutaneous melanoma. For example, the flow and timing domain of QUADAS-2 questions whether the time interval between the index test (SLNB) and the reference standard (clinical follow-up) might have introduced bias. If a study includes patients who have not been followed up for long enough for a false-negative result to declare itself, there is a risk that the study will miss false-negative results, underestimate the false-negative rate, and overestimate sensitivity and NPV. The follow-up duration of studies included in the 2011 meta-analysis^14^ ranged from 7 to 72 (median 32.8) months. For context, patients in the UK are generally followed up with clinical lymph basin examination for 5 years after SLNB^16^, and there are reports of false-negative results presenting after more than 8 years^17^. In keeping with this potential source of bias, metaregression in that sample of papers suggested that the longer the study follow-up, the higher the false-negative rate (*P* = 0.002)^13^.

It is not clear to what extent biases exist in previous estimates of the diagnostic test accuracy of SLNB, or whether they are clinically relevant. It is important and timely to revisit this, while the results of the KEYNOTE 716^18^ and CheckMate 76K^19^ trials suggest that adjuvant immunotherapy may have a role in reducing disease recurrence in patients with a negative SLNB, at the cost of adverse treatment effects,. Adjuvant immunotherapy carries a serious risk of irreversible side-effects. In a patient with stage IIB or IIC disease, the choice of either SLNB or proceeding straight to immunotherapy may be (partly) influenced by the NPV of the SLNB in that patient. Patients with a very low risk of a false-negative result may rather opt for SLNB and avoid the risks of immunotherapy in the event of a negative result. A patient with a higher risk of a false-negative result might have less faith in a negative result and be more willing to accept the risks of immunotherapy.

The objectives of this study were to estimate the sensitivity of SLNB for cutaneous melanoma based on studies that had followed up patients for at least a mean or median of 5 years, to systematically appraise the risk of bias in these studies according to QUADAS-2, and to provide NPV estimates across a plausible range of pretest probabilities for lymph node metastasis.

## Methods

This study was designed and reported in line with the PRISMA-DTA guidance^20,21^. AMSTAR 2 was employed to evaluate the quality of the study^22^. It was preregistered on the PROSPERO database (CRD42022371038).

### Eligibility criteria

Studies of SLNB for cutaneous melanoma were included in which sample size, number of positive results, number of false-negative results, and number of true-negative results were available or calculable. Retrospective case series, cohort studies, and randomized clinical trials were eligible study designs. The search was limited to English language studies.

The initial plan was to include only studies in which every patient had been followed up for a minimum of 5 years. However, this yielded only two studies and so screening was rerun to include studies with follow-up for a mean or median of 5 years. The consequences of this decision are discussed in the risk-of-bias section of the results.

### Search strategy

Search strategies were developed comprising indexed and free search terms (*supplementary methods*). Ovid MEDLINE and Embase databases were searched from inception to 28 May 2025. Abstracts and then full texts were screened independently and in duplicate through the Covidence platform, with a third author resolving conflicts.

### Data extraction

Two authors extracted data from included studies independently, with conflicts resolved by a third reviewer. Data items included: study author and year; study design; sample size; mean/median age; mean/median duration of follow-up; anatomical location of melanoma and SLNB; melanoma subtype; number of positive results; number of true-negative results; number of false-negative results; type of imaging; type of radioactive tracer; type of dye; histological technique; and adverse events.

### Risk of bias

The QUADAS-2 tool was used to assess risk of bias in sensitivity estimates. The items used are presented in the *supplementary methods*. This assessment was completed independently by two authors, with a third author resolving conflicts.

### Data synthesis

Sensitivity was estimated for each study. Sensitivity estimates were meta-analysed using the MetaDTA platform, which employs a random-effects bivariate binominal model. This is fitted as a generalized linear mixed-effects model with the lme4 R package^23^. Every positive result was assumed to be a true-positive (and therefore the specificity of SLNB in each case was 1.0). Confidence intervals were generated to the 95% level.

For each study, the prevalence (or pretest probability) of lymph node metastasis in patients undergoing SLNB was calculated. This was defined as the sum of true-positives and false-negatives, divided by the total sample size. These values were taken to represent a plausible range of pretest probabilities, and NPV was calculated across this range, as recommended by Trikalinos *et al.*^13^. Specifically, NPV was calculated as:


$$\frac{\mathrm{Specificity}\;\times \;(1-\mathrm{prevalence})}{\left(1-\mathrm{sensitivity})\times \mathrm{prevalence}+\mathrm{specificity}\times (1-\mathrm{prevalence}\right)},$$

assuming a specificity of 1.0.

## Results

**Fig. 1:**
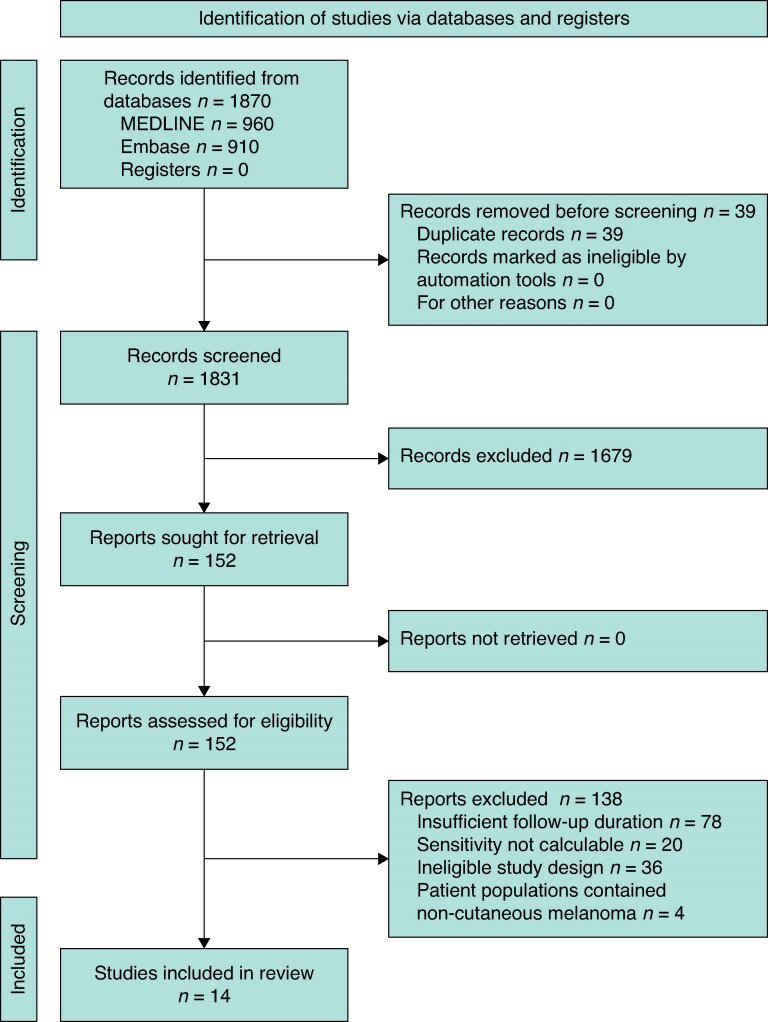
PRISMA flow diagram showing selection of articles for review

Following deduplication, 1705 studies were screened, with 14 meeting the full inclusion criteria (*Fig. 1*)^24^.

### Study characteristics

The 14 studies^9,17,25–37^ collectively included 8447 patients. Study characteristics are presented in *Table S1*. Although all studies employed preoperative lymphoscintigraphy and intraoperative tracing with ^99m^Tc and blue dye, the dose of ^99m^Tc and type of dye varied between studies. Where reported, all studies used haematoxylin and eosin staining and immunohistochemical analysis. Adverse events were reported in four studies.

### Risk of bias

**Fig. 2:**
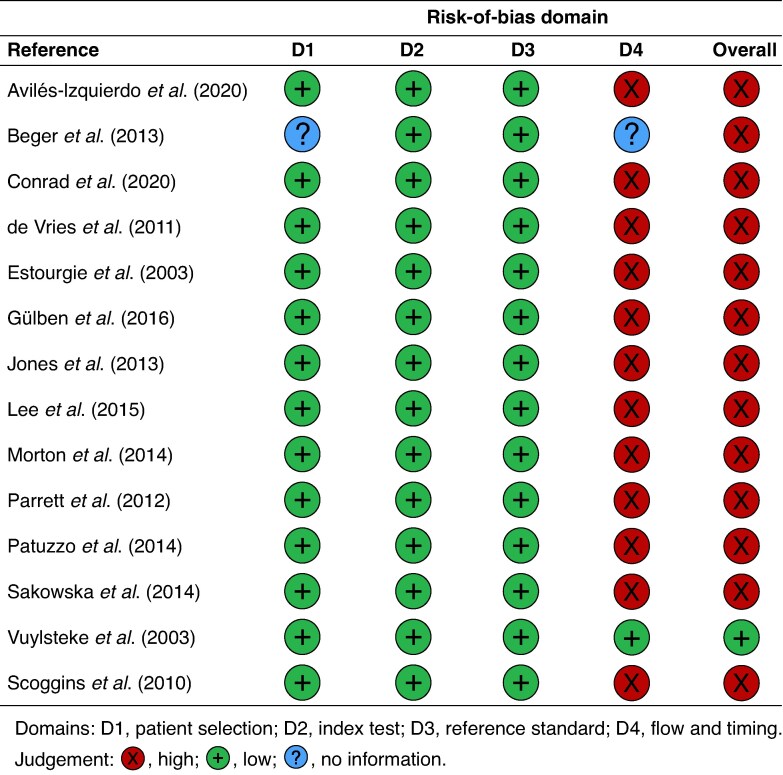
Risk-of-bias traffic light plot of included studies

Overall, 13 of the 14 studies had a high risk of bias, and this was largely due to the flow and timing of the study (*Fig. 2*). One study^26^ recruited only Caucasian patients, resulting in an unclear risk of bias in domain 1. One study^32^ did not standardize the SLNB technique, and included patients in whom only blue dye had been used. Two studies^9,33^ focused only on melanoma of the head and neck. All studies followed routine clinical follow-up as a reference standard, with a low risk of bias in domain 3; however, it is important to note that it is unclear how homogeneous follow-up practices were across studies. Only three studies^26,33,36^ followed every included patient for 5 years (a reference standard in line with national UK clinical guidance^16^), and only one study was considered to have a low risk of bias in domain 4. QUADAS-2 items are available in detail in *Table S2*. The exclusion of non-English language studies increased the risk of bias due to over-representing the results from English-speaking countries.

### Sensitivity and NPV

Studies generally did not present results with sufficient granularity for subgroup analysis by tumour stage, location, or histological subtype.

**Fig. 3:**
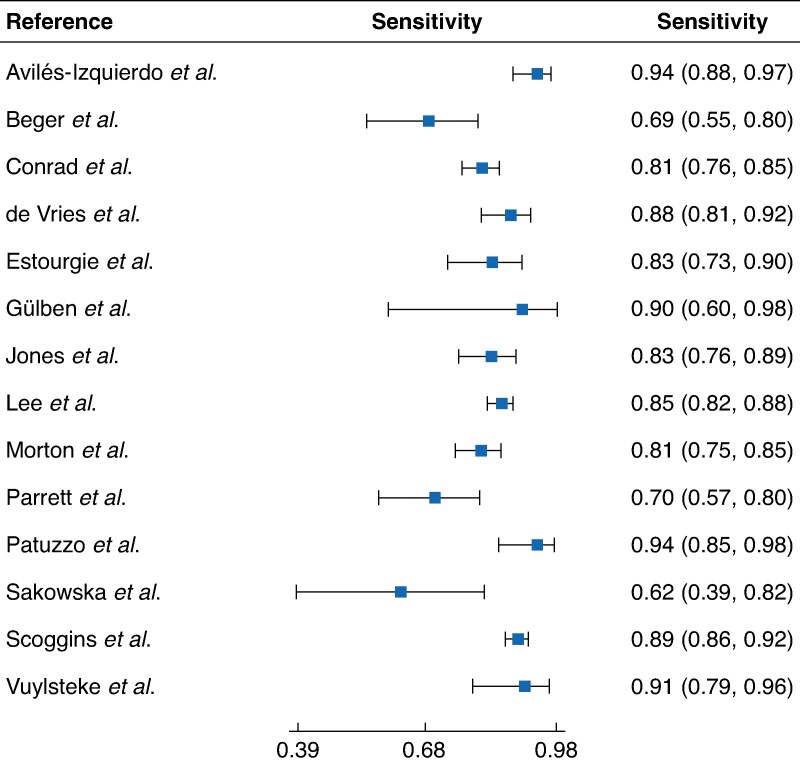
Forest plot of sensitivity estimates across studies Values are shown with 95% confidence intervals.

Overall sensitivity estimates are presented in *Fig. 3*. The pooled sensitivity estimate across studies was 0.85 (95% confidence interval (c.i.) 0.80 to 0.88). The prevalence of lymph node metastasis among studies ranged from 0.16 to 0.32 and this was taken to represent a plausible range of pretest probabilities for calculation of NPV.

**Fig. 4:**
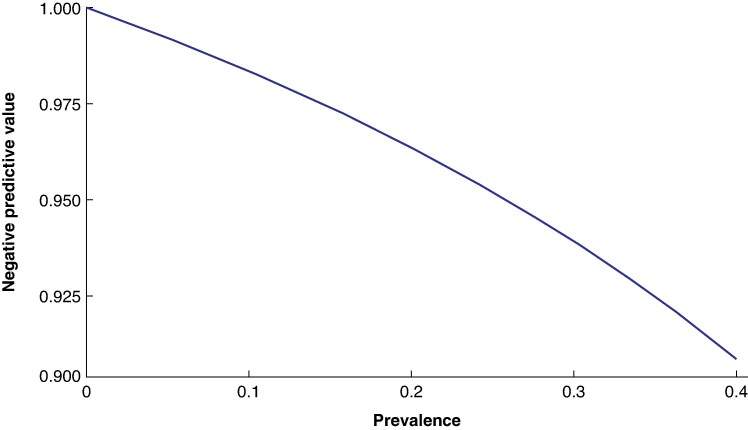
Negative predictive values across difference prevalence estimates (or pretest probabilities for a positive result)

*Figure 4* shows NPV estimates across the range of plausible prevalence estimates. According to these results, if a patient's pretest probability of lymph node metastasis is 0.16, the NPV of SLNB (probability that a negative result is true) is 0.97. If a patient's pretest probability of lymph node metastasis is 0.32, the NPV of SLNB is 0.93.

## Discussion

The sensitivity of SLNB was estimated across all types and anatomical locations of cutaneous melanoma as 0.85 (95% c.i. 0.80 to 0.88), and the NPV was estimated to fall between 0.93 and 0.97, depending on the pretest probability of a positive result. Previous work^38–42^ has focused on modelling the pretest probability of a positive SLNB result, based on patient and tumour characteristics. For example, the Melanoma Institute Australia nomogram^43^ can be used to estimate the pretest probability of a positive SLNB result based on age, tumour thickness, mitotic rate, histological subtype, ulceration status, and presence of lymphovascular invasion. There is also increasing interest in gene expression profiling as a potential tool for predicting the pretest probability of a positive SLNB. The NPV curve (*Fig. 4*) complements this work by allowing clinicians and patients to interpret a negative SLNB result in the context of the pretest probability. For example, if a patient's pretest probability of sentinel lymph node involvement is 0.20, and their SLNB result is negative, the patient can be assured that, based on studies with an average follow-up of over 5 years, the chance that this SLNB result is correct is over 0.96. Although the variation in NPV across plausible pretest probabilities is not large (0.93–0.97), a given patient's NPV can contribute to decisions about follow-up frequency. For example, a patient at the lower end of this spectrum (higher chance of false-negative result) who struggles with self-surveillance may particularly benefit from intensified follow-up.

It is recognized that the included studies demonstrated a risk of positive bias and, as such, the estimates of sensitivity and NPV are also at risk of positive bias. Previous sensitivity estimates for SLNB in cutaneous melanoma are mostly at risk of bias owing to duration of follow-up, and the inclusion of patients who may have false-negative results that are yet to be identified. Although there is a risk of bias in the literature, the meta-analysis of studies with at least a mean or median follow up of 5 years has produced sensitivity and NPV estimates that are only slightly lower than those reported previously, providing reassurance to clinicians and patients alike. Previous point estimates of NPV (which did not account for pretest probability) ranged from 0.94 to 0.99^4^. It is possible that biases introduced by studies with shorter follow-up times are not clinically significant, or that the study's limitations have introduced a comparable level of bias. Only one study in the pooled estimate had a low risk of bias. Because studies were included based on mean or median follow-up time, it is possible that some patients included in this estimate were followed up for short periods and had false-negative results that were yet to be discovered. Notably, the study^31^ rated as having a low risk of bias predicted a high sensitivity (0.91, 95% c.i. 0.79 to 0.96). Second, it is possible that reporting bias exists in the literature, particularly in case series. Providers with a high proportion of false-negative results may be reluctant to publish their findings, positively biasing pooled estimates of sensitivity and NPV further.

The generalizability of findings should be interpreted cautiously. The sensitivity and NPV of SLNB will vary with differing practices in radiology, surgery, and histopathology, and are likely to vary with tumour location and subtype. Owing to limitations in the granularity of published data, meaningful subgroup analyses were not possible. SLNB of the head and neck is generally considered a significantly different procedure from that in the groin or axilla. Melanoma of the head and neck has complex and variable lymphatic drainage^44^, and the technical demands of the procedure may lead to differences in sensitivity and NPV estimates. Two included studies^9,35^ focused solely on melanoma of the head and neck. Although these were not outliers in the forest plot (*Fig. 3*), there is a possibility of selection bias masking a lower sensitivity and NPV in this subgroup. A study^36,44^ reported a false-negative rate nearly three times higher in head and neck cutaneous melanomas compared with other body regions, although the median follow-up of patients in the study was not long enough to for it to be included in the meta-analysis. Potential variability in the index test (SLNB technique) and reference standard (clinical follow-up) must be acknowledged. Different units may have different protocols for performing the procedure, analysing the specimens, and follow-up. These could all affect the detection of false-negative results.

Future SLNB research would benefit from the availability of individual patient data (with appropriate ethical safeguards). This would permit subgroup analyses and further exploration of bias, and assist in the development and evaluation of clinical prediction models. National-level registries are now providing valuable data-driven insights in other surgical areas^45,46^, and frameworks that permit international collaborators to work on large, granular data sets have driven recent successes in epidemiological science, particularly surrounding the COVID-19 pandemic^47^. A suggestion is that this approach would drive advances in SLNB for melanoma here too. In the meantime, it is recommended that authors examining the diagnostic test accuracy of SLNB consider sources of bias, such as those outlined in QUADAS-2, when conducting and reporting their work. For example, in line with common follow-up practice, defining a false-negative result as a lymph node recurrence in a previously negatively sampled basin, and a true-negative result as a negatively sampled basin without recurrence over 5 years, is recommended. Reporting statistics such as sensitivity, rather than ‘false-negative rate’, is also recommended.

It is important to note that surgical and imaging approaches to SLNB are evolving, for example the emerging practice of sentinel lymph node mapping with superparamagnetic iron oxide^48^, indocyanine green^49^, or single-photo emission computed tomography with integrated computed tomography^50^. Future research that aims to assess the sensitivity of these techniques (or compare their sensitivity against preoperative lymphoscintigraphy and intraoperative tracing with ^99m^Tc and blue dye) should control for the pretest probability of a positive result in their analyses, and could apply the results reported here as a benchmark.

The sensitivity of SLNB for cutaneous melanoma was estimated as 0.85 (95% c.i. 0.80 to 0.88), and the NPV to lie between 0.93 and 0.97, depending on the pretest probability of a positive result. Existing estimates, including these, are at risk of positive bias, largely due to the inclusion of patients who may have unrecognized false-positive results. Despite this, SLNB is an excellent test to rule out lymph node metastasis. With the study's limitations in mind, clinicians can use the NPV curve to contextualize a negative SLNB result in the context of a patient's pretest probability. For example, if a patient returns to clinic after a negative SLNB result, and their pretest probability of lymph node metastasis was 0.16 as calculated by the Melanoma Institute Australia nomogram, the patient can be reassured that the literature pooled in this review suggests there is a 97% chance that their SLNB result is true.

## Supplementary Material

zraf089_Supplementary_Data

## Data Availability

The data underlying this article will be shared on reasonable request to the corresponding author.
