# Supplementary material for: Sensitivity and negative predictive value of sentinel lymph node biopsy for cutaneous melanoma for diagnosing nodal metastasis: meta-analysis of diagnostic test accuracy
Source: BJS Open. 2025 Aug 14;9(4):zraf089. doi: 10.1093/bjsopen/zraf089 (PMC12351453; doi:10.1093/bjsopen/zraf089)
Supplement: zraf089_Supplementary_Data [file zraf089_supplementary_data.zip › Supplementary_material.docx]

Sensitivity and negative predictive value of sentinel lymph node biopsy for cutaneous melanoma for diagnosing nodal metastasis: meta-analysis of diagnostic test accuracy

Sensitivity and NPV of SLNB for cutaneous melanoma: a meta-analysis

*Conrad Harrison^1,2,3^, *Samuel Willis^4^, Mary Rose Harvey^3^, Rakhshan Kamran^1^, Ryckie G Wade^5^, Thomas D Dobbs^4,6^, Oliver Cassell^2^

*Equal contribution

1. Nuffield Department of Orthopaedics, Rheumatology and Musculoskeletal Sciences, University of Oxford, Oxford, UK
2. Oxford University Hospitals, Oxford, UK
3. Oxford University Clinical Academic Graduate School, Oxford, UK
4. Swansea University Medical School, Swansea, UK
5. Leeds Institute for Medical Research, University of Leeds, Leeds, UK
6. Welsh Centre for Burns and Plastic Surgery, Morriston Hospital, Swansea, UK

**Corresponding author**

Thomas Dobbs

Welsh Centre for Burns and Plastic Surgery,

Morriston Hospital,

Swansea,

UK

[tomdobbs@doctors.org.uk](mailto:tomdobbs@doctors.org.uk)

ORCID ID: 0000-0002-5176-7741

**Supplementary Materials – Index**

**Supplementary Methods**

Supplementary Methods S1: Medline and Embase search strategy *page 3-4*

Supplementary Methods S2: QUADAS-2 items to assess bias in sensitivity estimates *page 5*

**Supplementary Results**

Supplementary Table S1: Characteristics of included studies. SLNB: sentinel lymph node biopsy. *page 6-9*

Supplementary Table S2: QUADAS-2 risk of bias assessment *page 10*

**Supplementary Methods**

**Supplementary Methods S1: Medline and Embase search strategy**

Medline search strategy

1. Sentinel Lymph Node Biopsy/

2. Sentinel Lymph Node/su [Surgery]

3. ("sentinel lymph node*" or "lymph node biops*" or SLNB*).ab,kw,ti.

4. 1 or 2 or 3

5. Melanoma/

6. "melanoma*".ab,kw,ti.

7. 5 or 6

8. "Sensitivity and Specificity"/

9. (sensitivit* or specificit*).ab,kw,ti.

10. False Negative Reactions/

11. "false negativ* ".ab,kw,ti.

12. "Predictive Value of Tests"/

13. "negative predictive".ab,kw,ti.

14. "negative value* ".ab,kw,ti.

15. ROC Curve/

16. ("receiver operating" or ROC).ab,kw,ti.

17. 8 or 9 or 10 or 11 or 12 or 13 or 14 or 15 or 16

18. 4 and 7 and 17

19. limit 18 to (english language and yr="1991 -Current")

Embase search strategy

1. Sentinel Lymph Node Biopsy/

2. Sentinel Lymph Node/su [Surgery]

3. ("sentinel lymph node*" or "lymph node biops*" or SLNB*).ab,kw,ti.

4. 1 or 2 or 3

5. Melanoma/

6. "melanoma*".ab,kw,ti.

7. 5 or 6

8. "Sensitivity and Specificity"/

9. (sensitivit* or specificit*).ab,kw,ti.

10. False Negative Reactions/

11. "false negativ* ".ab,kw,ti.

12. "Predictive Value of Tests"/

13. "negative predictive".ab,kw,ti.

14. "negative value* ".ab,kw,ti.

15. ROC Curve/

16. ("receiver operating" or ROC).ab,kw,ti.

17. 8 or 9 or 10 or 11 or 12 or 13 or 14 or 15 or 16

18. 4 and 7 and 17

19. limit 18 to (english language and yr="1991 -Current")

20. limit 19 to conference abstracts

21. 19 not 20

**Supplementary Methods S2: QUADAS-2 items to assess bias in sensitivity estimates**

| **Domain 1: Patient selection** |
| --- |
| Was a consecutive or random sample of patients enrolled? |
| Was a case-control design avoided? |
| Did the study avoid inappropriate exclusions? |
| Is there concern that the included patients do not match the review question? |
| **Domain 2: Index tests** |
| Was the SLNB technique described? |
| Was at least one form of imaging and at least one form of intraoperative tracing used to identify the sentinel node(s)? |
| Is there concern that the index test, its conduct or the interpretation differ from the review question? |
| **Domain 3: Reference standard** |
| Could the reference standard, its conduct, or its interpretation have introduced bias? |
| Was false negative clearly defined as a nodal recurrence in a basin which was previously sampled negatively? |
| **Domain 4: Flow and Timing** |
| Was there an interval of at least five years between SLNB and follow up, observed for every patient included in the analysis? |
| Were all patients included in the analysis (i.e. no patients were lost to follow up)? |
| Could the patient flow have introduced bias? |

SLNB: Sentinel Lymph Node Biopsy

**Supplementary Results**

**Supplementary Table S1. Characteristics of included studies. SLNB: sentinel lymph node biopsy.**

| Study | Study design | Sample size | Follow up (Months) | Age | Tumour location | SLNB location | Histological subtype | Radioactive tracer | Dye | Histological technique | Adverse events |
| --- | --- | --- | --- | --- | --- | --- | --- | --- | --- | --- | --- |
| Aviles-Izquierdo, 2020^25^ | Case series | 440 | Median 70, Range 12-240 | Median 56, Range 13-88 | 35 Head and Neck  205 Trunk  70 upper limb  130 lower limb | Not reported | 232 Superficial Spreading  128 Nodular  38 Acral-lentiginous  5 Lentigo maligna  37 Other | Technetium-99m (20-30MBq) | Methylene blue | H&E stain and immunohistochemical analysis | Not reported |
| Beger, 2013^26^ | Case series | 201 | Median 120 | Not reported | 4 Head and Neck  89 Trunk  31 Upper limb  77 Lower limb | 10 Head and Neck  133 Axilla  98 Groin  4 Popliteal | 96 Superficial Spreading  61 Nodular  11 Acral-lentiginous  3 Lentigo maligna  30 Other | Technetium-99m | Patent blue | H&E stain and immunohistochemical analysis | 4 Transient nerve lesions  3 lymphedema 3 wound infection  1 postoperative lymphocele |
| Conrad, 2020^27^ | Case series | 109 | Median 77, Range 9-204 | Mean 57, Range 13-87 | 20 Head and Neck  36 Trunks  10 Upper limb  25 Lower limb | 26 Head and Neck  51 Axilla  29 Inguinal | 23 Superficial Spreading  31 Nodular  5 Acral-lentiginous  5 Lentigo maligna  40 Other | Not reported | Not reported | Not reported | Not reported |
| De Vries, 2011^28^ | Case series | 450 | Median 64.8, Range 2.4- 173.8 | Median 53.2, Range 11-84 | Not reported | Not reported | 478 Superficial Spreading  290 Nodular  24 Acral-lentiginous  66 Other | Technetium-99m (40-60MBq) | Patent blue | H&E stain and immunohistochemical analysis | 2 anaphylaxis to dye  31 seroma |
| Estourgie, 2003^29^ | Case series | 250 | Median 72, Range 12.3- 104.4 | Mean 48, Range 16- 78 | 15 Head and Neck  95 Trunk  39 Upper limb  101 Lower limb | 25 Head and Neck  138 Axilla  133 Groin  29 Other | 140 Superficial Spreading  88 Nodular  10 Acral-lentiginous  2 Lentigo maligna  8 Other | Technetium-99m (65.5MBq) | Patent blue | H&E stain and immunohistochemical analysis | 1 Anaphylaxis to dye  5 wound infection  4 seroma  3 lymph fistula  2 neuropraxia  2 hematoma  10 lymphodema |
| Gülben. 2016^30^ | Case series | 40 | Median 70, Range 23- 168 | Mean 53, Range 24- 74 | 14 Axial  26 Extremities | Not reported | 23 Superficial Spreading  12 Nodular  5 Other | Technetium-99m (0.05-1mCi) | Isosulfane blue | H&E stain and immunohistochemical analysis | Not reported |
| Jones, 2013^17^ | Case series | 520 | Median 61, Range 1- 154 | Mean 49.7, Range not reported | 110 Head and Neck  205 Trunk  82 Upper limb  123 Lower limb | 117 Head and Neck  210 Axilla  150 Groin  43 Multiple | 190 Superficial Spreading  68 Nodular  29 Acral-lentiginous  233 Other | Technetium-99m | Methylene and Isosulfane blue | H&E stain and immunohistochemical analysis | Not reported |
| Lee, 2015^321^ | Case series | 2986 | Median 93, Range not reported | Mean 56, Range not reported | 617 Head and Neck  1157 Trunk  581 Upper limb  631 Lower limb | 1417 Axilla  780 Inguinal  766 Cervical  23 Other | Not reported | Technetium-99m | Lymphazurin | H&E stain and immunohistochemical analysis | Not reported |
| Morton, 2014^32^ | Randomized controlled trial | 938 | 10 years for all | Weighted mean 52.3 | Approximately 430 arm or leg and 510 other site | Not reported | Not reported | Not reported, some cases done with blue dye alone | Not reported | H&E stain and immunohistochemical analysis | Not reported |
| Parrett, 2012^9^ | Case series | 365 | Median 96, Interquartile range 42- 114 | Mean 60, Range 10-94 | 365 Head and Neck | 365 Head and Neck | Not reported | Technetium-99m | Isosulfan blue | H&E stain and immunohistochemical analysis | 3 facial nerve paresis of marginal mandibular branch  7 temporary spinal accessory nerve paresis  9 preauricular numbness  12 hematoma |
| Patuzzo, 2014^33^ | Case series | 331 | Median 62, Range 6- 156 | Median 60, Range 15- 88 | 331 Head and Neck | 331 Head and Neck | Not reported | Technetium-99m | Methylene blue | H&E stain and immunohistochemical analysis | Not reported |
| Sakowska, 2014^34^ | Case series | 95 | Median 65, Range 47- 112 | Median positive SLNB 63, Range 19-73  Median Negative SLNB 60, Range 20- 84 | 2 Head and Neck  29 Trunks  39 Upper limb  25 Lower limb | Not reported | Not reported | Technetium-99m | Not reported | H&E stain and immunohistochemical analysis | Not reported |
| Scoggins, 2009^35^ | Case series | 2451 | Median 61, Range not reported | Median 51, Range not reported | 1356 Axial  1089 Extremities | Not reported | 1145 Superficial spreading  1194 other | Technetium-99m | Not reported | H&E stain and immunohistochemical analysis | Not reported |
| Vuylsteke, 2002^36^ | Case series | 209 | Median 72, Range 60- 100 | Median 47, Range 18- 81 | 16 Head and Neck  83 Trunk  24 Upper limb  86 Lower limb |  | 146 Superficial Spreading  57 Nodular  3 Acral-lentiginous  3 Lentigo malgina | Technetium-99m (1.0mCi) | Patent blue | H&E stain and immunohistochemical analysis | Not reported |

**Supplementary Table S2: Summary of QUADAS-2 risk of bias assessments. Please contact the authors for a more detailed item-level assessment, if required.**

|  | Domain 1 | Domain 2 | Domain 3 | Domain 4 |
| --- | --- | --- | --- | --- |
| Aviles-Izquierdo et al. 2020 | Low | Low | Low | High |
| Beger et al. 2013 | Unclear | Low | Low | Unclear |
| Conrad et al. 2020 | Low | Low | Low | High |
| de Vries et al. 2011 | Low | Low | Low | High |
| Estourgie et al. 2003 | Low | Low | Low | High |
| Gülben et al. 2016 | Low | Low | Low | High |
| Jones et al. 2013 | Low | Low | Low | High |
| Lee et al. 2015 | Low | Low | Low | High |
| Parrett et al. 2012 | Low | Low | Low | High |
| Patuzzo et al. 2014 | Low | Low | Low | High |
| Sakowska et al. 2014 | Low | Low | Low | High |
| Vuylsteke et al. 2003 | Low | Low | Low | Low |
| Scoggins et al. 2010 | Low | Low | Low | High |
| Morton et al. 2014 | Low | Low | Low | High |
